# Long-term effects of percutaneous coronary intervention versus coronary artery surgery in elderly with multi-vessel coronary artery disease

**DOI:** 10.1186/s43044-022-00323-4

**Published:** 2022-12-28

**Authors:** Amr Kamal, Abdel Megeid Kandil, Mohamed Sadaka, Basem Ramadan

**Affiliations:** 1 Department of Cardiology and Angiology, Faculty of Medicine, Alexandria University, Champollion Street, Azareeta, Alexandria, Egypt; 2 Department of Cardiothoracic Surgery, Faculty of Medicine, Alexandria University, Alexandria, Egypt

**Keywords:** Percutaneous coronary intervention, Coronary artery disease, Coronary artery bypass, Stroke, Heart failure, Angiography, Hospitals

## Abstract

**Background:**

The most prevalent illness among the elderly is coronary artery disease (CAD), and most of this population present with multi-vessel CAD which constitutes a common management difficulty among elderly people. This study aimed to compare long-term consequences of percutaneous coronary intervention (PCI) versus coronary artery bypass graft (CABG) revascularization in elderly patients with multi-vessel coronary artery disease.

**Results:**

This retrospective study evaluated 100 elderly patients with multi-vessel CAD divided into two groups, group 1 the PCI group and group 2 the CABG group. The main findings of the study showed that CABG group had a longer hospital stay than the PCI group (8.16 vs. 2.02 days; *P* < 0.001). A considerably higher mean residual SYNTAX score was observed in the PCI group than CABG group which was 1.15 and 0.2, respectively (*p*-value < 0.001). The in-hospital major adverse cardiac events rate (MACE) in both groups was not statistically significant. Regarding the in-hospital mortality rate, although it was higher in the CABG group (6%) versus 2% in the PCI group, yet it was not statistically significant. The long-term MACE in this study revealed that 12.2% of PCI patients had heart failure compared to 6.4% in the CABG group, which was statistically insignificant. In the long-term follow-up, the revascularization rate of CABG group was higher than the PCI group; however, it was statistically insignificant. CABG group had a higher rate of stroke than PCI group being 4.3% and 2%, respectively; yet this difference was not statistically considerable. The long-term mortality rate among the PCI and CABG group was 10.2% and 4.3%, respectively.

**Conclusions:**

Elderly patients with multi-vessel CAD, PCI with stenting, and CABG were statistically equivalent in long-term death and MI rates, stroke, repeat revascularization. A non-statistically notable distinction between the two groups as regards MACE, composite of death or MI, and all-cause mortality was found. This may have implication on choice of management strategy among elderly patients with multi-vessel CAD.

## Background

The most prevalent illness among the elderly is coronary artery disease (CAD), which is also the predominant cause of fatality in elderly population for both sexes [1]. The increased aging led to increased incidence of CAD owing to increased oxidative stress and inflammatory of process which ultimately to vascular stiffness and endothelial dysfunction. Compared to younger adults, aging patients typically have more severe coronary atherosclerosis, in addition to having a higher incidence of left main coronary artery (LMCA) obstruction, multi-vessel disease (MVD), and prior myocardial infarction (MI) [2, 3].

The senior population’s growth rate has increased enormously and will continue to do so in the future. Typically, doctors have a tendency to perform less aggressive therapies and more conservative treatments. However, aged individuals are more likely to experience difficulties due to age-related comorbidity and physiological changes. The relative merits of percutaneous intervention (PCI) versus coronary artery bypass graft (CABG) surgery for treating individuals suffering from MVD, whether or not they also have distal left main coronary artery disease (LMCAD), are still up for debate [4, 5].

The balancing risks and benefits of revascularization in aged patients diagnosed with MVD is a challenge and the appropriate revascularization strategy is unclear. Other studies showed that there is no notable distinction between CABG and PCI in elderly patients having MVD in mortality. Elderly patients' ideal option for coronary revascularization is largely unknown. Although there are many clinical studies and assessment system about indication and contraindication of revascularization, targeted studies and point scoring system in elderly patients are relatively absent; however, our country and international community are facing aging of population, associated with increased morbidity of elderly CAD patients. Thus, it is necessary to enhance our knowledge about the outcome of the retrospective study of PCI against CABG in aged patients to provide targeted assessment system for elderly CAD patients [6]. This study aims to compare and analyze the long-term consequences of revascularization through percutaneous coronary intervention (PCI) versus coronary artery bypass grafting (CABG) in elderly patients having multi-vessel coronary artery disease (MVD).

## Methods

A retrospective study conducted on the files of at least 100 elderly patients of 65 years or more diagnosed as multi-vessel CAD revascularized either with CABG or PCI from January 2015 till December 2019 in Alexandria university hospital and International Cardiac Center. Two groups of the patients were formed, namely group 1 and group 2; each had 50 patients where the former group underwent PCI, and the later CABG. All patients’ files will be reviewed as regards the weight and height. By collecting history with special insistence on gender, age, family history of CAD, history of CAD risk factors as D.M., hyperlipidemia, hypertension, cigarette smoking, major acute cerebrovascular, cardiac, and ischemic cardiac incidents, data of some laboratory investigation such as hemoglobin, urea, creatinine and lipid profile were retrieved from patients’ files. Records of electrocardiogram (ECG) and echocardiogram were also documented.

Records of coronary angiography will be revised as: Syntax scores I and II, number of defective vessels, presence of LMCAD, number of lesions, residual syntax score after PCI, access site, vessels approached, number of stents, intraprocedural complications, and procedural outcomes.

Regarding CABG, the following was documented from patients’ records; number of grafts, type of grafts, intraprocedural complications, post-procedural complications, and procedural outcomes.

The patient’s files of both groups will be reviewed as in-hospital and long-term follow-up care of MACE including MI, vessel revascularization or non-target vessel revascularization, bleeding, heart failure, stroke, and death.

### Statistical data analysis

IBM SPSS software package version 20.0 was applied to evaluate the data (Armonk, NY: IBM Corp). Number and percentage were used to analyze the qualitative data. The Kolmogorov–Smirnov test was used to evaluate the normality of the distribution. The mean, range (minimum and maximum), median, standard deviation (SD), and interquartile range (IQR) were utilized to characterize the quantitative data. The 5% significance level was used to establish the collected results’ importance. The tests that were used are:

1. Chi-square test: Categorical variables were compared among various groups.
2. Monte Carlo correction or Fisher’s exact: This test was done when more than 20% of cells had an expected count lower than 5 to rectify the Chi-square test.
3. Student’s *t* test: Two groups under study were compared for quantitative variables having normal distributions.
4. Mann–Whitney *U* test: For comparing, two studied groups were compared having quantitative variables with abnormal distribution.

## Results

The main demographic data and risk factors are summarized in Tables 1 and 2, respectively. The clinical presentation of the patients varied from exertional dyspnea, angina, and MI in both groups. The frequency of exertional dyspnea, angina and MI in PCI group was 24%, 54%, and 22%, respectively, while that of the CABG group was 24%, 56%, and 20%, respectively (Table 3). The coronary angiographic findings are given in Table 4.

**Table 1:** Two studied groups compared with reference to demographic data

Demographic data	Group 1 (*n* = 50)		Group 2 (*n* = 50)		Test of sig.	*P*
	No.	%	No.	%		
*Gender*						
Male	38	76.0	42	84.0	*χ*^2^=1.000	0.317
Female	12	24.0	8	16.0		
*Age (years)*						
Min.–Max	65.0–79.0	65.0–74.0			*t* = 1.434	0.155
Mean ± SD	68.44 ± 3.93	67.50 ± 2.45				
Median (IQR)	67.0 (66.0–71.0)	67.0 (66.0–69.0)				
*Height (cm)*						
Min.–Max.	145.0–196.0	150.0–186.0			*t* = 0.794	0.429
Mean ± SD	171.70 ± 9.09	170.36 ± 7.73				
Median (IQR)	174.0 (165.0–178.0)	170.0 (165.0–176.0)				
*Weight (kg)*						
Min.–Max.	65.0–140.0	64.0–110.0			*U* = 1103.50	0.310
Mean ± SD	87.12 ± 12.10	88.15 ± 10.32				
Median (IQR)	85.50 (80.0–92.0)	90.0 (80.0–95.0)				
*BMI*						
Min.–Max.	24.98–44.44	23.23–42.19				
Mean ± SD	29.61 ± 3.97	30.46 ± 3.85			*t* = 1.085	0.281
Median (IQR)	26.68 (27.1–31.2)	30.28(27.8–32.3)				

*χ*^2^ Chi-square test, *t* Student *t* test, *U* Mann–Whitney test, *p p*-value for comparison between the two studied groups, *Group 1* Patient treated with PCI and drug-eluting stents, *Group 2* Patient treated with CABG

**Table 2:** Two studied groups compared with respect to history of CAD risk factors

History of CAD risk factors	Group 1 (*n* = 50)		Group 2 (*n* = 50)		*χ*^2^	*P*
	No.	%	No.	%		
DM	31	62.0	37	74.0	1.654	0.198
Hyperlipedemia	18	36.0	16	32.0	0.178	0.673
Hypertension	37	74.0	31	62.0	1.654	0.198
*Cigarette smoking*						
No	32	64.0	39	78.0	3.871	^MC^*p* = 0.120
Yes	18	36.0	10	20.0		
Ex-smoker	0	0.0	1	2.0		

*χ*^2^ Chi-square test, *MC* Monte Carlo, *p p*-value for comparison between the two studied groups, *Group 1* patient treated with PCI and drug-eluting stents, *Group 2* patient treated with CABG

*Statistically significant at *p* ≤ 0.05

**Table 3:** Two studied groups compared in accordance with clinical presentation

Clinical presentation	Group 1 (*n* = 50)		Group 2 (*n* = 50)		*χ*^2^	*p*
	No.	%	No.	%		
Exertional dyspnea	12	24.0	12	24.0		
Angina	27	54.0	28	56.0	0.066	0.968
MI	11	22.0	10	20.0		

*χ*^2^ Chi-square test, *p p*-value for comparison between the two studied groups, *Group 1* patient treated with PCI and drug-eluting stents, *Group 2* patient treated with CABG

**Table 4:** Two studied groups compared in accordance with coronary angiography data

Coronary angiography	Group 1 (*n* = 50)		Group 2 (*n* = 50)		*χ*^2^	*p*
	No.	%	No.	%		
*Presence of left main disease*						
Absent	46	92.0	25	50.0	21.418*	< 0.001*
Present	4	8.0	25	50.0		
*Number of diseased vessels*						
LAD (and diagonal) lesion						
Free	4	8.0	3	6.0	0.154	^FE^*p* = 1.000
Yes	46	92.0	47	94.0		
*LCX (and OM) lesion*						
Free	12	24.0	8	16.0	1.000	0.317
Yes	38	76.0	42	84.0		
*RCA (PDA and PL) lesion*						
Free	15	30.0	7	14.0	3.730	0.053
Yes	35	70.0	43	86.0		
*RAMUS lesion*						
Free	48	96.0	45	90.0	1.382	^FE^*p* = 0.436

Number of lesions	No.	%	No.	%	Test of sig.	*p*
2	11	22.0	2	4.0	*χ*^2^ = 26.956	^MC^*p* < 0.001*
3	24	48.0	13	26.0		
4	12	24.0	11	22.0		
5	1	2.0	12	24.0		
6	2	4.0	5	10.0		
7	0	0.0	4	8.0		
8	0	0.0	2	4.0		
9	0	0.0	1	2.0		
Min.–Max	2.0–6.0	2.0–9.0			*U* = 574.0*	< 0.001*
Mean ± SD	3.18 ± 0.94	4.60 ± 1.62				
Median (IQR)	3.0 (3.0–4.0)	4.0 (3.0–5.0)				
*Syntax score I*						
< 22	37	74.0	24	48.0	*χ*^2^ = 8.138*	^MC^*p* = 0.017*
22–32	12	24.0	20	40.0		
> 32	1	2.0	6	12.0		
Min.–Max	10.0–35.50	11.0–67.0	U = 825.0*	0.003*		
Mean ± SD	19.06 ± 5.66	23.72 ± 9.11				
Median (IQR)	18.25 (14.0–22.0)	22.0 (18.0–28.0)				
*Syntax score II*						
PCI	2	4.0	2	4.0	*χ*^2^ = 1.678	^MC^*p* = 0.437
PCI or CABG	26	52.0	32	64.0		
CABG	22	44.0	16	32.0		
Min.–Max	21.30–56.0	18.10–45.70			*U* = 1166.0	0.562
Mean ± SD	32.06 ± 7.82	30.67 ± 5.42				
Median (IQR)	31.05 (26.0–36.50)	30.10 (27.40–32.70)				
*Euro score II*						
Min.–Max	–	0.64–3.35				
Mean ± SD	–	1.71 ± 0.61	–	–		
Median (IQR)	–	1.61 (1.23–2.17)				

*χ*^2^ Chi-square test, *FE* Fisher exact, *MC* Monte Carlo, *U* Mann Whitney test*, p p*-value for comparison between the two studied groups, *Group 1* Patient treated with PCI and drug-eluting stents, *Group 2* Patient treated with CABG

*Statistically significant at *p* ≤ 0.05

The current study showed that the frequency of left main lesion, LAD, LCX, RCA, and RAMUS lesions in PCI group were 8%, 92%, 76%, 70%, and 4%, respectively, while that of CABG were 50%, 94%, 84%, 86%, and 10%, respectively. According to our patients' angiographic characteristics, the mean number of lesions was 3.18 in the PCI group and 4.6 in the CABG group, respectively. With a *p*-value less than 0.001, the CABG group had a significantly higher mean number of lesions than the PCI group. The CABG group's SYNTAX score I was 23.72, considerably greater than the PCI group's 19.5, with a 0.003 *p*-value. In the CABG group, the Euro score was 1.71.

The CABG group had a considerably longer hospital stay duration than the PCI group (8.16 vs. 2.02 days; *P* < 0.001). With a *p*-value less than 0.001, the mean residual SYNTAX score was 1.15 in the PCI group and 0.2 in the CABG group, respectively.

Tables 4 and 5 show, respectively, the in-hospital and long-term follow-up of MACE in the PCI group and the CABG group. The in-hospital MACE in both the PCI group and the CABG group was as follows: the frequency of occurrence of in-hospital heart failure in the PCI group was 0% than 2% in the CABG group, while that of the renal impairment and myocardial infarction was 2% in both groups. Neither groups required revascularization nor had in-hospital bleeding.

**Table 5:** Two studied groups compared with respect to in-hospital MACE

In-hospital MACE	Group 1 (*n* = 50)		Group 2 (*n* = 50)		*χ*^2^	FEp	Follow-up MACE				*χ*^2^	FEp
	No.	%	No.	%			Group 1 (*n* = 49)		Group 2 (*n* = 47)			
							No.	%	No.	%		
*Heart failure*												
No	50	100.0	49	98.0	43	87.8	44	93.6	0.970	0.487	1.010	1.000
Yes	0	0.0	1	2.0	6	12.2	3	6.4				
*Renal impairment*												
No	49	98.0	49	98.0	44	89.8	45	95.7	1.256	0.436	0.0	1.000
Yes	1	2.0	1	2.0	5	10.2	2	4.3				
*Death*												
No	49	98.0	47	94.0	49	100	47	100	–	–	1.042	0.617
Yes	1	2.0	3	6.0	0	0.0	0	0.0				
*Myocardial infarction*												
No	49	98.0	49	98.0	49	100.0	46	97.9	1.054	0.490	0.0	1.000
Yes	1	2.0	1	2.0	0	0.0	1	2.1				
*Targeted vessel revascularization or non-target vessel revascularization*												
No	50	100	50	100.0	48	98.0	45	95.7	0.389	0.613	–	–
Yes	0	0.0	0	0.0	1	2.0	2	4.3				
*Stroke*												
No	49	98.0	48	96.0	49	100	47	100	–	–	0.344	1.000
Yes	1	2.0	2	4.0								
*Bleeding*												
No	50	100.0	50	100.0							–	–

*χ*^2^ Chi-square test, *FE* Fisher exact, *p* *p*-value for comparing between the two studied groups, *Group 1* Patient treated with PCI and drug-eluting stents, *Group 2* Patient treated with CABG

Regarding the fatality rate, although it was more in the CABG group with 6% versus 2% only in the PCI group, it failed to achieve statistical importance.

The long-term follow-up care of MACE in this study revealed that 12.2% of PCI patients had heart failure compared to 6.4% in the CABG group, which was statistically insignificant.

We assessed the mortality rates in long-term follow-ups among the PCI and CABG which was 10.2% and 4.3%, respectively.

However, we did not report any case of post-procedural myocardial infarction among both groups.

In the current study, in long-term follow-ups, the revascularization rate was greater among CABG patients than PCI patients, but this difference was statistically insignificant. (2.1 in CABG group % vs. 0% in PCI group).

The CABG group had a greater cerebrovascular stroke frequency compared to the PCI group being 4.3% in the former and 2% in the latter, but this difference was not statistically important.

Finally, we reported that none of our cases during the long-term follow-ups experienced bleeding neither in the CABG nor the PCI groups.

## Discussion

The angiographic characteristics of our patients pointed that the mean number of lesions were 3.18 and 4.6 in PCI and GABG group, respectively. With a less than 0.001 *p*-value, the CABG group had a considerably greater number of lesions than PCI group. The SYNTAX score I was 19.5 in the PCI group and 23.72 in the CABG group, which is significantly higher in the latter group with a 0.003 *p*-value. The Euroscore II was 1.71 in the CABG group. When comparing our results with the literature available we found accordance with the NOBLE, SYNTAX and Freedom trials as their SYNTAX score and number of lesions and Euro scores. Their SYNTAX score in PCI group was 22.5, 28.4, and 26.2, respectively, compared to 22.4, 29, and 26.1 in the CABG group. In the same trials, the mean number of lesions in the PCI group was 2, 4.3, and 5.65 compared to 2, 4.4, and 5.74 in the CABG group, respectively. The Euro scores in the CABG were 2, 3.8, and 2.8, respectively [7, 8]. With a mean value of 2.46, the number of stents used in the present research ranged from 1 to 5. The intraprocedural complications in the angiography were only in 2% of cases. These findings were comparable with the published trials, where the mean number of stents used in BEST trial [9], Freedom follow on study [10], and Gimbel et al. [11] study were 3.4, 3.4, and 1.71, respectively.

One of the major factors that affect the choice of the revascularization technique is the in-hospital stay. Comparing the CABG group to the PCI group, the hospital stay duration was noticeably longer in the CABG group (8.16 vs. 2.02 days; *P* < 0.001).

This finding comes in agreement with NOBLE trial that stated that the duration of indexed treatment admission in PCI group was 2 days compared to 9 days in CABG group (2 vs. 9 days; *P* < 0.001) [8]. Moreover, the CARDIA trial also confirmed the median time spent in CABG was significantly longer than PCI, being 9 days for the earlier and 1 day for the latter, with a *P*-value less than 0.001 [12].

Other studies observed a longer duration stays such as LE MANS trial and Becher et al., where the LE MANS trial, pinpointed that the mean days of hospitalization was 6.8 and 12.04 days in the PCI and CABG group, respectively, with *P*-value = 0.0007. Becher et al. reported that the CABG group’s hospital stay was substantially longer than the PCI group’s (16.1 vs. 8.8 days; *P* < 0.0.5) [14].

In the present study, we compared the in-hospital MACE in both the PCI and the CABG group. The PCI group’s frequency of occurrence of in-hospital heart failure was 0% compared to 2% in the CABG group, while that of the renal impairment and myocardial infarction was 2% in both groups. Neither groups required revascularization, nor had in-hospital bleeding.

The CABG group had a higher frequency of cerebrovascular stroke as compared to that of the PCI group being 4% in the former group and 2% in the latter group but was not statistically significant.

This could be attributed to elder and seriously ill population that is considered appropriate to undergo CABG. The ascending aorta's formation of atheromatous fragments during hypo-perfusion and surgical manipulation are the two factors that contribute to the etiology of cerebrovascular stroke in CABG [15].

The handling of the aorta in cannulation, anastomosis, and cross‐clamping of a conduit could represent a risk for thromboembolic process, where most patients undergoing CABG suffer from atherosclerosis of the ascending aorta and surgical manipulation can lead to detachment of atheromatous plaques or calcified deposits, specifically during the aortic cross‐clamp placement or removal. Immediate postoperative causes of cerebrovascular stroke in CABG patients are mainly due to atrial fibrillation (POAF) before hospital discharge. Also, previous causes mentioned in the intraoperative period may still cause embolic clots which lead to stroke. Additionally, in the early postoperative period, anesthetic residual effects can prevent the detection of intraoperative strokes. Another potential factor is the low cardiac output syndrome, prolonged inotrope usage, and postoperative bleeding, which is linked to hypo-perfusion caused by hypovolemia and often anemia which raise the risk of cerebrovascular stroke in these patients [16].

Regarding the fatality rate although it was more in the CABG group (6%) than 2% only in the PCI group yet it was statistical insignificant, which could be explained by the higher risk the patients are subjected to during such a major invasive operation and it might have reached the significance threshold if the sample size was increased.

Hsu et al. investigated the in-hospital MACE and compared it in both the CABG and PCI groups. They demonstrated that the frequency of in-hospital death was higher among CABG group (20.5%) than the PCI group (5%). There was a higher occurrence of in-hospital cerebrovascular stroke in the CABG group than PCI group being 2.6% in the former and 0% in the latter group. The repeated revascularization was observed in 2.6% of CABG patients and none in the PCI group.

Hsu et al. [17] justified these findings by the differences in the study population, where the CABG group was at high surgical risk based on the Euro score classification, which rationalizes the relatively high rate of observed adverse events.

In accordance with our outcomes Prashanth et al. describes similar frequencies of in-hospital MACE, where the frequencies of in-hospital death, MI and revascularization of targeted vessel in PCI patients were 2%, 2.9%, and 0%, respectively, compared to our results 2%, 2%, and 0%, respectively [18].

The long-term follow-up care of MACE in this study revealed that 12.2% of PCI patients had heart failure compared to 6.4% in the CABG group; however, it was statistically insignificant. Wang et al. reported that the patients undergoing PCI and CABG had heart failure frequency of 1.8 and 1.94, respectively, in the follow-up study [19].

We assessed the mortality rates in long-term follow-up care among the PCI and CABG group, which was 10.2% and 4.3%, respectively. The updated Noble reported comparable data as the rate of mortality in both the PCI and CABG groups were 9%. They concluded that although mortality was similar, yet patients who received PCI had higher myocardial infarction rates and repetition of revascularization, and recommended improving the long-term outcomes by tailoring patient selection and optimization [20].

On the other hand, the Freedom follow on the study pinpointed that the PCI group’s long-term mortality was greater compared to the CABG group with 24.3% versus 18.3% stating that compared to PCI-DES, CABG continues to reduce all-cause mortality better. The better survival with CABG has been attributed in part to the increasing use of internal mammary grafts in these trials [10].

In the current study, no case of post-procedural myocardial infarction among both groups was observed. Similarly, the EXCEL trial, 5-year follow-up reported the primary composite rate of MI in PCI and CABG group to be 5.1% and 2.4%, respectively [21].

Moreover, the PRECOMBAT extended study reported comparable results with slightly higher frequencies in PCI and CABG group (3.2 vs. 2.8). They attributed the lower MI rates in their research to adopting the strictest definition of MI available [22].

However, other studies revealed different results; the updated NOBLE study reported the frequency of post-procedural myocardial infarction among the PCI and CABG was 8% and 3%, respectively [20].

In long-term follow-up, the revascularization rate was greater among the CABG as compared to PCI in the current study but this increase was statistically insignificant (2.1 in CABG group % vs. 0% in PCI group). This could be attributed to proper selection of patient candidate to PCI and the highly efficient drug-eluting stents used nowadays. Moreover, a possible explanation of our results relay on the fact that the PCI patients had lower frequencies of LMCAD, lower SYNTAX score and lesser number of presenting lesions than the CABG group.

This finding is contradicting that published. Gimbel et al. reported target revascularization rate of and 5.8% and 12.5% in and CABG and PCI group, respectively, owing to commonly occurring unsuccessful revascularization (restenosis) following PCI than following CABG (graft failure) or by more complete revascularization following CABG than following PCI. Nevertheless, it is speculated that full revascularization is not necessary in these older patients to provide a decent long-term prognosis [11].

In the long-term follow-up of PCI patients compared to CABG, a higher risk of recurrent revascularization may be due to complicated anatomy and contrast agent limitations in PCI.

Moreover, the EXCEL trial reported similar findings in long-term follow-up of PCI and CABG regarding repeated revascularization being 11.6% and 5.8% in PCI and CABG groups, respectively. It is worth mentioning that most of revascularization was repeated by PCI procedures. Nevertheless, repeated revascularization is more likely correlated with MI and death [21].

As expected, the cerebrovascular stroke frequency was greater among the CABG than the PCI group being 4.3% in the former and 2% in the latter, yet this distinction was statistically insignificant.

This result is concurrent with multiple clinical studies such as CARDIA, PRECOMBAT extended, EXCEL follow-up, FREEDOM, and BEST trials, where the frequencies of cerebrovascular stroke in PCI group were 0.4, 1.9, 0.5, 2.4, and 2.5, respectively, while those of the CABG group were 2.8, 2.2, 0.8, 5.2, and 2.9, respectively [9, 12, 21–23].

These frequencies are slightly less in the PCI group as compared to the CABG group, yet some did not achieve the statistically significant level too.

The ascending atherosclerosis in the aorta could be related with the long-term incidence of cerebrovascular stroke post-CABG. Moreover, increased risk of factors in the aging process might be a possible explanation of cerebrovascular disease in CABG patients. Among the major risk factors, stroke are advanced age, previous stroke, previous stenosis of carotid artery, and previous PVD increased operation time and postoperative atrial fibrillation [24, 25]. The delayed factors associated with increased occurrence of stroke in CABG patients include decreased cardiac output, atrial fibrillation, myocardial infarction, and thrombophilia. Carotid stenosis, hypertension, peripheral vascular disease, diabetes mellitus, recent myocardial infarction, and/or renal failure are among the conditions that put patients at an elevated stroke risk following cardiac surgery.

Another possible etiological factor is association with unstable angina which could be attributed to the ongoing chronic inflammatory process in such patients as supported by the active inflammatory process in conjunction with building up of macrophages at the sites of ruptured plaques and consequently leading to increased thrombosis [26].

Furthermore, other studies showed a contradicting result. They stated that the cerebrovascular stroke was higher in the PCI group compared with the CABG group. Martins et al., NOBLE, and updated NOBLE are among these studies. The rate of occurrence of stroke in the PCI among these groups were 3.2%, 5%, and 4%, respectively, compared to 1.8%, 2%, and 4% in the CABG group, respectively [20, 27].

They explained such findings owing to lowering of repeat revascularization rate in the CABG group, as the repetition technique is linked to poor prognosis. They added that this might be partly justified by stent restenosis, incomplete revascularization in more complex cases. Another possible cause is that after 1 year, there is occurrence of cerebrovascular stroke in the PCI group, overlapping the stoppage of dual antiplatelet therapy inhibition. Yet, the decreased incidence of cerebrovascular stroke could be due chance [8, 27].

Finally, we reported that none of our cases in the long-term follow-up care experienced bleeding neither in the CABG nor the PCI groups. This could be explained by the advancement in performances in both procedures and skillful heart teams and proper management protocols applied in both techniques.

By exploring available literature, we noted an increased bleeding rate among CABG group than the PCI group (3.1% in CABG group versus 0.7% in PCI group) in the research study performed by Gimbel et al. [11].

The best trial too revealed that the fatal bleeding rate among the CABG and PCI group was 1.6% and 0.7%, respectively, while bleeding frequency with reference to thrombolysis in myocardial infarction (TIMI) was 6.8 in the PCI and 29.9 in the CABG group. CABG group’s remarkably higher rate is mostly attributed to an effect of the operation [9].

In conclusion, in the current retrospective study, no statistically considerable distinction between PCI and CABG was found concerning long-term follow-up incidence rates of MACE, heart failure, stroke composite of death, or MI, or among elderly patients having MVD. As a result, in long-term follow-up, the use of drug-eluting stents (DES) was not equivalent to CABG in terms of significant adverse cardiac events.

Furthermore, PCI can provide a substitute and safe approach to CABG in properly selected patients. However, CABG seems to provide more complete revascularization as denoted by the residual SYNTAX.

The ultimate medical judgment should be designated on a person’s baseline characteristics incorporating all the circumstances, taking in consideration life expectancy and quality of life.

The present study had inadequate statistical strength to permit a solid deduction, and further investigation is indispensable in this field.

## Conclusions

To conclude elderly patients diagnosed with MVD, PCI with stenting and CABG were related to statistically equivalent long-term MI and death, stroke, and repeated revascularization rates. The incidence of MACE, MI, stroke, composite of death, and all-cause mortality did not differ statistically among PCI and CABG.

## Data Availability

Data sharing is not applicable to this article as no datasets were generated or analyzed during the current study.

## Abbreviations

CAD: Coronary artery disease
PCI: Percutaneous coronary intervention
CABG: Coronary artery bypass graft
MVD: Multi-vessel disease
MACE: Major adverse cardiac events
LMCA: Left main coronary artery
ECG: Electrocardiogram
